# A 27-Year Report from the Central Eye Bank of Iran: A complete translation from Farsi

**DOI:** 10.18502/jovr.v15i2.6731

**Published:** 2020-04-06

**Authors:** Mohammad Ali Javadi, Mozhgan Rezaei Kanavi, Sare Safi

**Affiliations:** ^1^ Ophthalmic Research Center, Shahid Beheshti University of Medical Sciences, Tehran, Iran; ^2^ Central Eye Bank of Iran, Tehran, Iran; ^3^ Ocular Tissue Engineering Research Center, Shahid Beheshti University of Medical Sciences, Tehran, Iran; ^4^ Ophthalmic Epidemiology Research Center, Shahid Beheshti University of Medical Sciences, Tehran, Iran

**Keywords:** Central Eye Bank of Iran, Corneal Transplants Per Capita, Descemet Stripping Automated Endothelial Keratoplasty, DSAEK; Penetrating Keratoplasty

## Abstract

**Purpose:**

To report the 27-year statistical data from the Central Eye Bank of Iran (CEBI) and its activity.

**Methods:**

All CEBI records regarding procured eyes, tissue utilizations, corneal transplants per capita, and indications for keratoplasty from 1991 to 2017 were analyzed.

**Results:**

In total, 115,743 whole eyes were donated during the 27-year period. Out of the 114,169 eyes donated between 1994 and 2017, 95,314 eyes were distributed for transplantation, and 95,057 corneas were actually transplanted. The mean annual rate of corneal transplants per capita was 55.10 ${}^{-6}$ ± 27.10 ${}^{-6}$ . Although penetrating keratoplasty (PKP, 70%) was the most common technique of corneal transplantation during the study period, it exhibited a decreasing trend between 2006 and 2017 (*P* = 0.048). It was in contrast to Descemet stripping automated endothelial keratoplasty (DSAEK) that demonstrated an increasing trend during the same period (*P* < 0.001). Keratoconus (KCN, 39.70%) was the most leading indication for keratoplasty over the last three decades followed by bullous keratopathy (BK, 18.5%), corneal scar and opacities (15.7%), and graft failure (GF, 7.5%), with an increasing trend for BK, GF, and KCN. A majority of scleral tissues (83.7%) were utilized for orbital implant protection.

**Conclusion:**

An increasing trend in the number of procured eyes was observed over the past 27 years in Iran. The most leading indications for corneal transplantation were KCN and BK. While PKP was the most common keratoplasty technique, DSAEK showed an increasing trend over the last 12 years.

##  INTRODUCTION

Corneal blindness is the third leading cause of avoidable visual impairment worldwide after cataract and glaucoma.^[1]^ Corneal transplantation via the restoration of visual function improves both the health status and the quality of life of patients undergoing keratoplasty.^[2]^ Leading indications for keratoplasty and implemented surgical techniques vary from one country to another depending on their geographic regions, socioeconomic conditions, and adaptation to recent surgical technological advancements.^[3,4,5,6,7,8,9,10,11]^ For instance, in developed countries, bullous keratopathy (BK) and Fuchs' endothelial dystrophy (FED) are the leading indications for keratoplasty, and with the adaptation of endothelial keratoplasty (EK) techniques in these countries, Descemet stripping automated endothelial keratoplasty (DSAEK) and Descemet membrane endothelial keratoplasty (DMEK) are the procedures of choice to selectively replace the diseased corneal endothelium.^[4,5,6,7,8,11]^ However, the leading indications for keratoplasty in developing countries vary from keratoconus (KCN) in Iran and Zimbabwe^[3,12,13]^ to infectious keratitis and corneal scarring in China and India.^[14,15]^ Moreover, penetrating keratoplasty (PKP) is still the most common technique for corneal transplantation in these countries.^[3,12,13][14,15]^

The Central Eye Bank of Iran (CEBI), the main eye bank in the center of the national corneal transplantation network over the last 27 years in Iran, has been processing and distributing the tissues for corneal and scleral transplantations in accordance with the international medical standards.^[16]^ Herein, we report a 27-year statistical data and present a comprehensive picture of eye banking activity at the CEBI.

##  METHODS

After obtaining full approval from the ethics committee of the Ophthalmic Research Center, affiliated with Shahid Beheshti University of Medical Sciences in Tehran, Iran, a retrospective study was conducted to review and analyze all the compiled eye bank data between 1991 and 2017 at the CEBI. The data compiled included annual and total rates of procured eyes (1991–2017), ocular tissue utilizations (2006–2017), corneal transplants per capita (2006–2017), indications for keratoplasty (1994–2017), and post-transplantation adverse reactions (2006–2017). Detailed data regarding postoperative adverse reaction reported to the CEBI were not available before 2006. All the statistical analyses were performed using the Statistical Package for the Social Sciences (SPSS) software version 22 (SPSS, Inc., Chicago, IL, USA). Linear regression models were utilized to investigate any changing trend in the variables of interest. *P*-value < 0.05 was considered statistically significant.

##  RESULTS

### Eye Procurement

**Figure 1:**
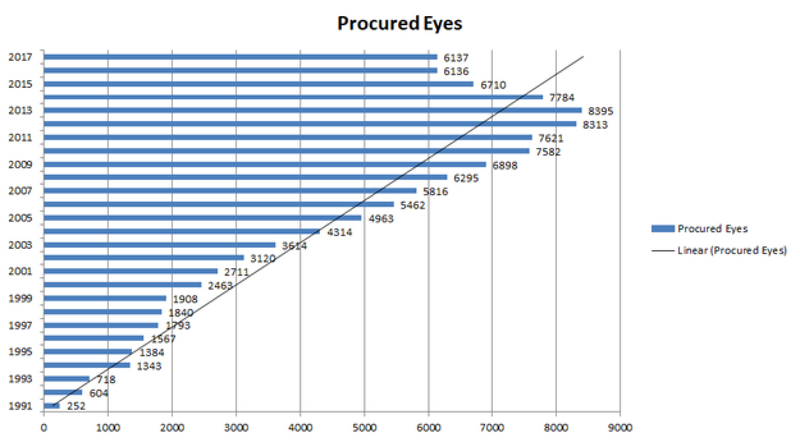
The annual rates of corneal procurement in Iran. Note the increasing rate of corneal procurement between 1991 and 2017.

During the 27-year period, a total of 115,743 whole eyes from 58,804 donors were procured. The annual rates of corneal procurement are illustrated in Figure 1, showing an increasing rate over the specified period excluding the last three years in which the corneal procurement rate showed a moderate reduction. Considering that detailed data for 1,574 eyes (from 787 donors) procured between 1991 and 1993 were not available, the analysis of the majority of the variables was not possible for this certain time period. Between 1994 and 2017, a total of 114,169 eyes from 58,017 donors were procured. Donors' age ranged from 1 month to 85 years, and 79.4% were male. The majority of donors were in the age range of 21–40 (43.1%) and 41–60 (37.7%) years.

### Ocular Tissue Distribution and Utilization

**Figure 2:**
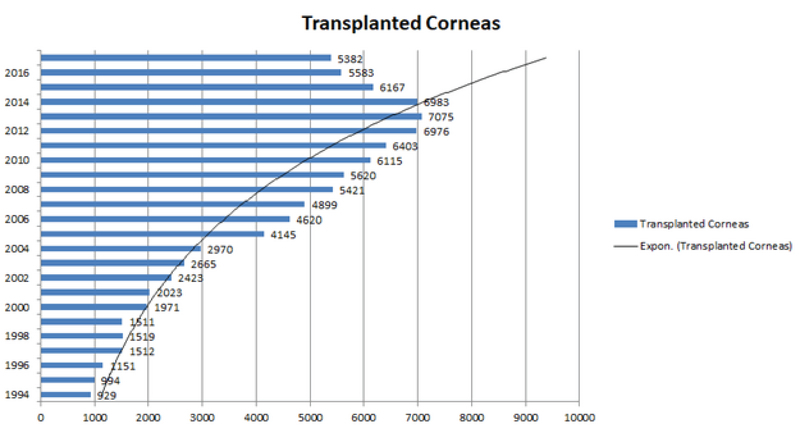
The annual rate of keratoplasties in Iran. Note the increasing rate of corneal transplantation between 1994 and 2016.

Among the 114,169 eyes procured between 1994 and 2017, 95,314 (83.5%) eyes were distributed for transplantation purposes, and 95,057 corneas were transplanted. Small numbers of the procured eyes that were ineligible for transplantation were distributed and utilized for research purposes (933, 0.8%). The rate of keratoplasties per year is shown in Figure 2, illustrating an increasing rate over the specified period. The distributed corneas were either in the form of excised corneoscleral discs (84.6%) maintained in cold storage media such as Optisol GS ${}^{\mathrm{TM}}$ and Eusol C ${}^{\mathrm{TM}}$ or as whole globes (17.4%) preserved in cold moist chambers. Moreover, 7.2% of the whole eyes had been frozen in –70°, and their corresponding defrosted corneas were transplanted. Out of the distributed globes/corneas, 257 (0.3%) cases were returned non-transplanted to the CEBI due to pre- or intraoperative technical errors at the time of surgery.

**Figure 3:**
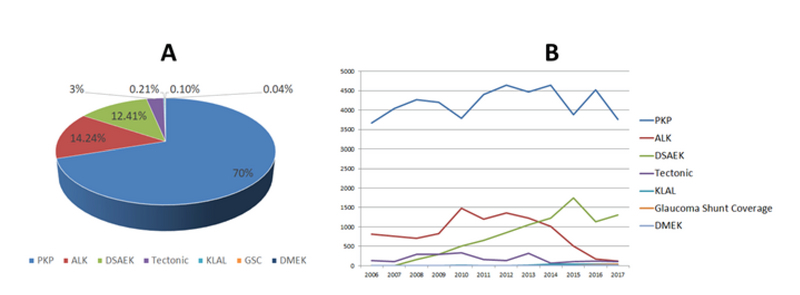
The mean rate of transplantation procedures and their corresponding relative trends. (A) penetrating keratoplasty was the most common keratoplasty procedure (70%) over the last 12 years, followed by anterior lamellar keratoplasty (14.24%), Descemet stripping automated endothelial keratoplasty (12.41%), tectonic (3%), keratolimbal allografts (0.21%), glaucoma shunt coverage (0.10%), and Descemet membrane endothelial keratoplasty (0.04%). (B) The relative trends of corneal transplantation techniques between 2006 and 2017 were illustrated.ALK, anterior lamellar keratoplasty; DMEK, Descemet membrane endothelial keratoplasty; DSAEK, Descemet stripping automated endothelial keratoplasty; KLAL, keratolimbal allografts; GSC, glaucoma shunt coverage; PKP, penetrating keratoplasty.

**Figure 4:**
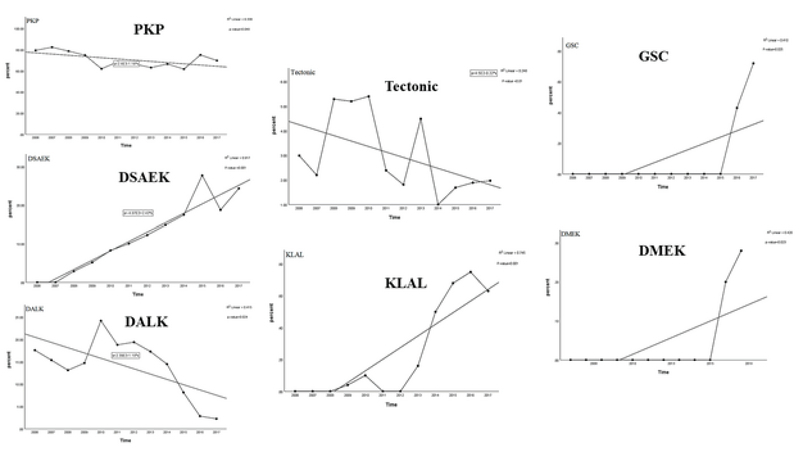
Trends of keratoplasty techniques from 2006 to 2017 in Iran. A significant decreasing trend for the rates of penetrating keratoplasty (*P* = 0.048), anterior lamellar keratoplasty (*P* = 0.024), and tectonic surgery (*P* = 0.01) is observed. A significant increasing trend of change for the rates of Descemet stripping automated endothelial keratoplasty (*P* < 0.001), keratolimbal allografts (*P* < 0.001), glaucoma shunt coverage (*P* = 0.025), and Descemet membrane endothelial keratoplasty (*P* = 0.023) since their introduction is observed. The regression R ${}^{2}$ measures the goodness of fit of the regression line. DMEK, Descemet membrane endothelial keratoplasty; DSAEK, Descemet stripping automated endothelial keratoplasty; KLAL, keratolimbal allografts; GSC, glaucoma shunt coverage; PKP, penetrating keratoplasty.

PKP and similar procedures such as tectonic surgery and anterior lamellar keratoplasty accounted for all transplantation techniques used before 2006 with no exact data on each procedure. Between 2006 and 2017, 71,796 corneas were utilized for keratoplasty. The rate of each transplantation technique and its corresponding relative trend over the last 12 years are illustrated in Figure 3. Over this period, PKP was the most common technique of corneal transplantation (70%), followed by anterior lamellar keratoplasty (ALK, 14.24%), DSAEK (12.41%), and tectonic surgery (3%). A significant decreasing trend was observed in the rates of PKP (*P* = 0.048), ALK (*P* = 0.024), and tectonic surgery (*P* = 0.01), whereas DSAEK demonstrated an increasing trend (*P* < 0.001) over the last 12 years [Figure 4]. A small number of corneas were utilized for keratolimbal allografts (KLALs, 0.21%), glaucoma shunt coverage (GSC, 0.10%), and DMEK procedure (0.04%) with an increasing trend since the introduction of the corresponding procedures (*P* < 0.001 for KLAL, *P* = 0.025 for GSC, and *P* = 0.023 for DMEK) [Figure 4]. Almost all the KLALs performed during the study period utilized donor limbal tissues for recipients with primary or secondary limbal stem cell deficiency (LSCD) of variable etiologies, among which chemical burn injuries and mustard gas keratopathies predominated.

There were no specified data regarding the utilization of scleral tissues before 2006. From 2006 to 2017, out of the 83,149 procured donated eyes, 2,212 (2.7%) scleral tissues preserved in absolute alcohol were utilized to provide an additional protection in the orbital implants following enucleation (83.7%) and for glaucoma shunt patching (16.3%).

### Corneal Transplants Per Capita

**Figure 5:**
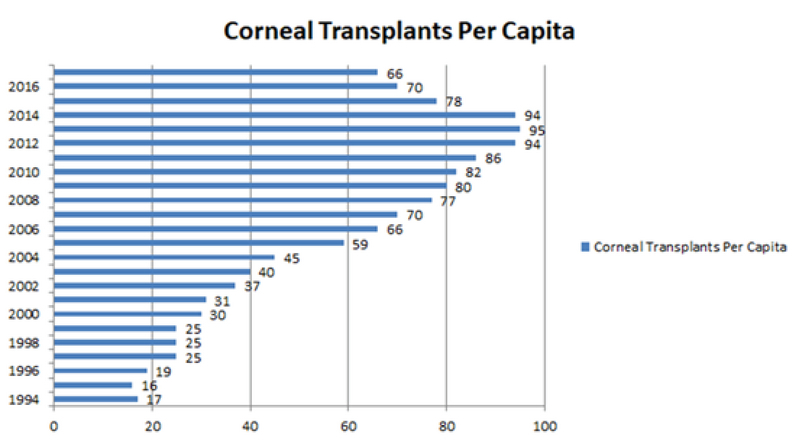
The annual rates of corneal transplants per capita in Iran. Note an increasing rate of corneal transplants per capita from 1994 to 2017, ranging from 16.10 ${}^{-6}$ in 1995 to 95.10 ${}^{-6}$ in 2013.

The calculated transplantation rate per million inhabitants in Iran is shown in Figure 5. The annual numbers of corneal transplants per capita over the last 24 years ranged from 16.10 ${}^{-6}$ to 95.10 ${}^{-6}$ (mean, 55.3.10 ${}^{-6}$ ± 27.10 ${}^{-6}$ ). This increasing rate of corneal transplants per capita paralleled the increase in the Iranian population over the specified period.

### Indications for Corneal Transplantation

**Figure 6:**
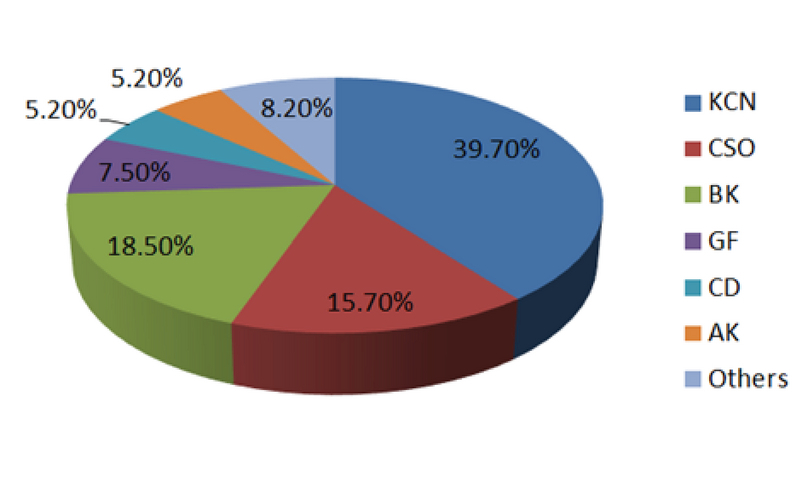
Mean rates (%) of indications for keratoplasty procedures utilizing corneas provided by the Central Eye Bank of Iran. Keratoconus has been the most common indication for keratoplasty (39.70%) over the last 24 years, followed by bullous keratopathy (18.5%), corneal scar and opacities (15.7%), graft failure (7.5%), corneal dystrophies (5.20%), active keratitis (5.20%), and others (8.2%). AK, active keratitis; BK, bullous keratopathy; CD, corneal dystrophies; CSO, corneal scar and opacities; GF, graft failure; KCN, keratoconus.

**Figure 7:**
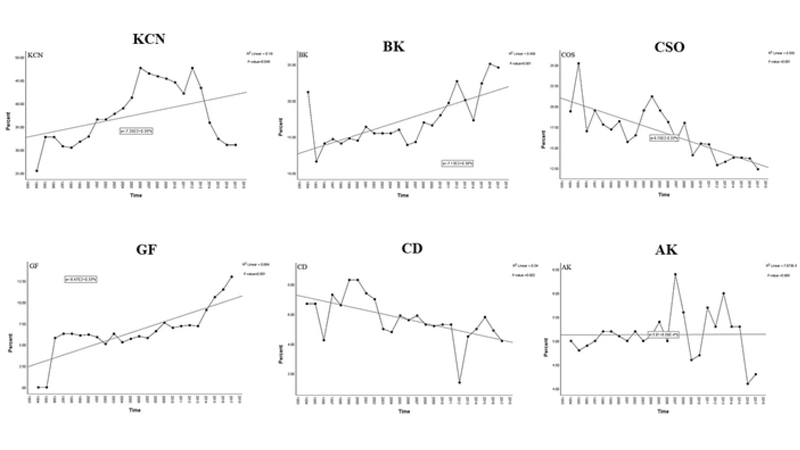
Trends of the leading indications for keratoplasty in Iran over the last 24 years. The trend of change for the rates of bullous keratopathy (*P* < 0.001), graft failure (*P* < 0.001), and keratoconus (*P* = 0.046) shows a significant increase. A significant decreasing trend is observed for the rates of corneal scar and opacities (*P* < 0.001) and corneal dystrophies (*P* = 0.002). No significant change of trend is noted for active keratitis (*P* = 0.968). The regression R ${}^{2}$ measures the goodness of fit of the regression line. AK, active keratitis; BK, bullous keratopathy; CD, corneal dystrophies; CSO, corneal scar and opacities; GF, graft failure; KCN, keratoconus.

Figures 6 and 7 illustrate the indications for keratoplasty and their corresponding trends between 1994 and 2017, respectively. The donors were predominantly male (61.2%) and aged from 6 months to 96 years. The majority of donors were between 21 and 40 years (38.2%), followed by those between 61 and 80 years (24.1%) and between 41 and 60 years (20.3%). KCN was the main leading indication for corneal transplantation (39.70%) followed by BK (18.5%), corneal scar and opacities (CSO, 15.7%), graft failure (GF, 7.5%), corneal dystrophies (CD, 5.20%), and active keratitis (AK, 5.20%). The remaining indications (8.2%) which were considered “others” included chemical burn injuries, LSCD, primary glaucoma tube shunt coverage, exposed glaucoma tube shunts, leaking filtering bleb, and cases with no specific diagnosis.

A significant increasing trend for BK (*P* < 0.001), GF (*P* < 0.001), and KCN (*P* = 0.046) was observed; however, a decreasing trend for CSO (*P* < 0.001) and CDs (*P* = 0.002) over the last 24 years was observed. AK revealed no significant change in the trend during the specified period (*P* = 0.968). The four most leading CDs were macular corneal dystrophy (MCD, 2.79%), FED (1.28%), congenital hereditary endothelial dystrophy (CHED, 0.44%), and granular corneal dystrophy (0.41%). Out of the 8,950 patients undergoing endothelial keratoplasty (8923, DSAEK; 27, DMEK), 78.7% had BK after cataract surgery, and 21.3% had either FED or CHED. All the DMEK surgeries utilized pre-stripped DMEK tissues that were prepared at the CEBI.

### Ocular Tissues Used for Glaucoma Shunt Patching

Between 2006 and 2017, sclera preserved in absolute alcohol was the most frequently used tissue for glaucoma shunt patching; however, in the last two years, frozen corneal tissues (in –70°C) that had unsuitable endothelial quality for keratoplasty were limitedly used as alternatives to scleral tissues for patching glaucoma shunts.

### Adverse Reactions

Based on the eye bank postoperative reports over the past 12 years, out of the 71,796 transplants performed in Iran, adverse reactions were reported in 63 cases (0.088%). Furthermore, 84.2% of all transplanted corneas were used for PKP/ALK, and 12.5% were used for EK. The adverse reactions were reported in 26 (0.04%) and 37 (0.41%) cases after PKP/ALK and EK, respectively. The most common adverse reactions after EK were related to the surgery (*n* = 22, 0.24%) followed by primary GF (*n* = 15, 0.17%). The most frequently reported adverse reactions after PKP/ALK were primary GF (*n* = 13; 0.022%) followed by infectious keratitis (*n* = 7, 0.012%) and endophthalmitis (*n* = 6, 0.01%). The microbiologic pathogens in cases with transmission of infection were fungi (57%) and Gram-negative organisms (43%). Microbiologic pathogens were isolated in 83.3% of endophthalmitis cases, among which Gram-positive organisms (60%) were the most common retrieved microorganisms.

##  DISCUSSION

Increased number of corneal transplants in Iran performed from 1991 to 2017 reflects an expanding activity of the CEBI and associated cornea surgeons. The annual rate of corneal transplants per capita varied considerably over the last three decades in Iran. The 27-year statistics of the CEBI with a well-organized eye banking infrastructure shows a mean annual rate of 55.3.10 ${}^{-6}$ corneal transplants per capita, which is comparable with the annual rates of keratoplasty per capita in developed countries such as France (59.2.10 ${}^{-6}$ ), Germany (54.0.10 ${}^{-6}$ ), and the UK (61.3.10 ${}^{-6}$ ).^[18]^ This probably reflects the CEBI's ability to immediately supply corneal tissues according to the nation's demands. Moreover, it is worthy to know that Iran is one of the self-sufficient countries among the 148 countries, with satisfactory access to donated corneas for transplant, without the need to import corneal tissues.^[18]^

Although PKP is the most common keratoplasty technique in our series, it showed a decreasing trend over the last decade. This is probably attributed to the increasing rate of DSAEK performed for BK cases that showed a dramatic increase during the specified period. These results are consistent with those reported by the Eye Bank Association of America (EBAA) in 2016^[16]^ in which EK remained the top leading keratoplasty technique in the USA. Similarly, the New Zealand National Eye Bank reported decreasing trend of PKP and increasing trend of DSAEK almost at the same time period.^[19]^

Although KCN, BK, CSO, GF, CDs, and AK are the main six indications for corneal graft in our series, the map of some indications has changed over the past three decades. Consistent with our prior published report,^[3,12]^ indications for keratoplasty in Iran are characterized by a larger proportion of KCN, but a lower frequency of AK and CDs. Moreover, BK, GF, and KCN showed a significantly increasing trend over the last 24 years. Increasing trend of BK could be explained by the widespread application of phacoemulsification for cataract surgery in Iran,^[20]^ and the facts that the surgery was performed even in patients with low corneal endothelial cell counts ^[21,22,23]^ and by less experienced surgeons.^[24]^

GF revealed an increasing trend over the past three decades, which was consistent with the increased number of patients undergoing corneal transplantation and concurrent with the widespread expansion in the rate of keratoplasties in Iran. The increasing rate of GF can also be attributed to the increasing rate of EK techniques and the decreasing interest in performing ALK techniques in Iran. GF, defined as a loss of graft clarity or refractive quality, is mainly observed as a result of allogeneic immune rejections. However, nonimmune factors such as glaucoma, endothelial cell failure, aging, and viral and nonviral infections may cause GF.^[25,26,27]^ Although GF was reported as the most leading indication for keratoplasty in the UK three decades ago,^[28]^ it did not remain on the top and declined to the third place in the later decade, which could be due to the significant increase in the number of ALK operations performed.^[29]^

KCN in our series ranked the first place among the leading indications for keratoplasty in Iran and showed an increasing trend up to 2012. However, as explained in our prior report,^[3]^ a downward trend was observed after 2012, which could be due to the increasing rate of implementation of collagen corneal cross-linking and intracorneal rings in the moderate form of KCN. Moreover, the overshadowing effect of the increasing trends of BK and GF over the last decade is considered another probable explanation.

CSO, in the current study, demonstrated a significant decreasing trend over the last 24 years. Based on our prior reports,^[3,12]^ CSO was replaced by BK and declined from the second to the third place, which can be partially overshadowed by the increasing trend of BK. However, in some developing countries, CSO caused by infectious keratitis and trauma was considered as the most common indication for keratoplasty.^[14,30,31]^ Factors reducing the incidence of infectious keratitis, such as public awareness about the importance of early diagnosis and proper management of infectious keratitis and preventive measures,^[30]^ may play significant roles in the decreasing trend of CSO in Iran.

Different from some Western countries where FED is the most common indication for keratoplasty,^[16,32]^ CDs along with AK ranked the fifth among the leading indications for keratoplasty in Iran over the last three decades. Moreover, consistent with prior studies,^[3,12]^ FED in our series was the second common indication for keratoplasty after MCD among the different types of CDs. High prevalence of aging population in Western countries and the increasing awareness regarding the benefits of the triple procedure employing EK techniques in cataractous cases with advanced FED may explain this difference.^[8,33]^ Different from our prior published reports in which no significant change of trend in the number of procured eyes was shown, our present series showed a significant decreasing trend over the last three decades, which can be explained by the difference in the surveyed time periods. However, there is a possibility that the decreasing trend of CDs might have been overshadowed by the increasing trend of BK and GF. Regarding AK, our series did not show significant change of trend over the last three decades; this result is similar to that of our previous reports.^[3,12]^ Different from Iran and several developed countries where AK is not a common indication for corneal transplantation,^[7,16]^ AK is the most common indication for keratoplasty in the developing countries in Asia. This difference may be explained by the presence of large agricultural communities with poor farm and work safety standards and limited medical services, leading to higher prevalence of AK in these developing countries.^[9,34,35]^

Despite it was anticipated that ALK would replace PKP in corneal pathologies with normal endothelium,^[6]^ our series revealed a significant decreasing trend for ALK over the past 12 years; hence, ALK decreased to its lowest rate in 2017. This can be explained by the decreasing rate of transplantation for KCN after 2012. Another possible reason is that some surgeons may not have reported ALK in their postoperative reports. To prevent some postoperative complications due to the transplantation of low-quality grafts (e.g., persistent epithelial defects, graft edema, and suture-related complications), some surgeons prefer to report they perform PKP when actually deep anterior lamellar keratoplasty is performed to receive a donor tissue with good quality. This issue needs a comprehensive strategic planning to address both the eye bank and the surgeons' concerns.

Our series demonstrated a significant decreasing trend for tectonic graft for the past 12 years. Tectonic graft, considered as a challenging and emergent corneal transplantation, is performed in corneal pathologies that threaten the globe integrity such as infectious or noninfectious keratitis. This reinforces the critical role CEBI has in supplying donor corneas for tectonic purposes to retain corneal integrity and save the eye in such patients. The rate of tectonic graft in our series (3%) was significantly lower than the rate (11.4%) reported in a six-year analysis of the UK Transplant Registry.^[36]^ This difference can be explained by the different time intervals in which the surveys were performed; the UK Transplant Registry analysis has been performed between 1999 and 2005^[36]^ with no updated analysis on the data after 2005, whereas our survey analyzed the data on transplantation techniques after 2006.

There has been an increasing trend for new transplantation techniques such as KLAL, GSC using corneal tissue, and DMEK since their introduction in Iran. Therefore, CEBI has been providing donated corneal tissues for these techniques of transplantation since 2009. In our series, almost all the KLALs were performed in patients with LSCD predominantly caused by chemical burns. Although the management of chemical burn-induced ocular surface disorders is significantly challenging, with the recent development in ocular surface reconstruction and the current use of translational medicine such as cultured autologous limbal epithelial transplantation for this purpose in Iran,^[39,40]^ a decreasing rate of KLAL is anticipated over time.

GSC using donor corneal tissue has been implemented for the last two years in Iran, aiming to cover glaucoma shunt tubes primarily or manage the exposed tubes and leaking filtering blebs. It was well demonstrated that early surgical management of glaucoma tube-related complications reduces the risk of bleb-associated endophthalmitis.^[41]^ The use of donor corneal tissues for GCS is not only a therapeutic option but also provides excellent cosmetic results.^[42]^ Although the number of GSC surgeries was small, it revealed a significant increasing trend over the last two years. DMEK using CEBI-prepared pre-stripped tissues was implemented in a few tertiary eye centers in Iran in 2016 and, despite having a small proportion, demonstrated an increasing trend since then. Different from the USA in which DMEK is more popular than DSAEK,^[18]^ DMEK has still failed to gain popularity in Iran. This can be explained by the easier handling of the DSAEK lenticules as compared to delicate DMEK tissues. Moreover, the long learning curve for DMEK might have limited the use of this surgical technique by cornea surgeons in Iran. However, preparation of pre-stripped ready-to-use DMEK tissues with healthy corneal endothelium and use of user-friendly DM delivery systems to simplify the DMEK technique may encourage some cornea surgeons to substitute DSAEK with DMEK.

Over a 12-year period, the scleral tissues of 2.7% of the donated whole eyes were processed at the CEBI and distributed for cosmetic or emergent eye conditions, predominantly for wrapping the orbital implants after enucleation. With the emergence of frozen corneas for covering the glaucoma tube shunts since 2016, some glaucoma surgeons use these tissues rather than scleral tissue to achieve optimum cosmetic results.^[42]^

In our series, the overall rate of adverse reactions reported to the CEBI (0.088%) was significantly low and even less than the reported rate in the USA (1.2%).^[44]^ Considering that we analyzed only the officially reported adverse reactions encountered during early postoperative period, one of our main drawbacks in this part is probably the insufficient evidence on the adverse reactions that were not reported to the CEBI and the lack of a transplant registry report. In our analysis, the reported adverse reactions after EK, similar to the US report,^[44]^ was more frequent than those after PKP/ALK surgeries. This can be explained by long learning curves required for DSAEK and DMEK. Similar to the report by the EBAA between 2007 and 2014,^[45]^ primary GF predominated in our series over the last 12 years. Different from the EBAA report in which Candida species was the most frequent isolated pathogen in post-corneal graft endophthalmitis,^[45]^ Gram-positive microorganisms predominated in our series. Regarding infectious keratitis, no significant difference was found between the rate of fungal keratitis and keratitis caused by Gram-negative bacteria in our survey; this result is different from that of the EBAA report.^[45]^

The present survey demonstrated a partial reduction in the rate of corneal procurement from the CEBI after 2015, which was concurrent with launching a couple of minor eye banks in Tehran and Shiraz.^[3]^ Nevertheless, the activity of these minor eye banks in the national corneal transplantation network was not remarkable, and they had no roles in preparation of pre-cut DSAEK lenticules and pre-stripped DMEK tissues.

In conclusion, the CEBI has shown an expanding activity over the last 27 years, in a way that Iran has become one of the self-sufficient countries in the world that does not need to import corneal tissues. The low rate of reported adverse reactions indicated a relatively high safety of the graft system from donor harvesting and preparation in the CEBI to graft transplantation. With the emergence of EK techniques over the last decade in Iran, there has been an increasing trend in the rates of these surgical procedures and a decreasing trend of PKP. Despite this observation, PKP remained the most frequent transplantation procedure over the last 12 years. Although KCN was the most leading indication for keratoplasty in Iran, BK and GF showed an increasing trend over the last 24 years. Eye bank preparation of pre-cut thin lenticules of high endothelial quality for DSAEK was encouraging for the cornea surgeons to substitute PKP with DSAEK in cases with corneal endothelial diseases. It is anticipated that providing pre-stripped and pre-loaded DM tissues with healthy endothelial cells for DMEK may motivate the surgeons to gradually substitute DMEK for DSAEK.

##  Financial Support and Sponsorship

Nil.

##  Conflicts of Interest

There are no conflicts of interest.
